# A Novel Chemically Modified Curcumin “Normalizes” Wound-Healing in Rats with Experimentally Induced Type I Diabetes: Initial Studies

**DOI:** 10.1155/2016/5782904

**Published:** 2016-04-13

**Authors:** Yazhou Zhang, Steve A. McClain, Hsi-Ming Lee, Muna S. Elburki, Huiwen Yu, Ying Gu, Yu Zhang, Mark Wolff, Francis Johnson, Lorne M. Golub

**Affiliations:** ^1^Department of Oral Biology and Pathology, School of Dental Medicine, Stony Brook University, Stony Brook, NY 11794, USA; ^2^Department of Cariology and Comprehensive Care, College of Dentistry, New York University, New York, NY 10010, USA; ^3^Departments of Dermatology and Emergency Medicine, Stony Brook University, Stony Brook, NY 11794, USA; ^4^Department of General Dentistry, School of Dental Medicine, Stony Brook University, Stony Brook, NY 11794, USA; ^5^Department of Pharmacological Sciences, Stony Brook University, Stony Brook, NY 11794, USA

## Abstract

*Introduction*. Impaired wound-healing in diabetics can lead to life-threatening complications, such as limb amputation, associated in part with excessive matrix metalloproteinase- (MMP-) mediated degradation of collagen and other matrix constituents. In the current study, a novel triketonic chemically modified curcumin, CMC2.24, was tested for efficacy in healing of standardized skin wounds in streptozotocin-induced diabetic rats. Initially, CMC2.24 was daily applied topically at 1% or 3% concentrations or administered systemically (oral intubation; 30 mg/kg); controls received vehicle treatment only. Over 7 days, the diabetics exhibited impaired wound closure, assessed by gross and histologic measurements, compared to the nondiabetic controls. All drug treatments significantly improved wound closure with efficacy ratings as follows: 1% 2.24 > systemic 2.24 > 3% 2.24 with no effect on the severe hyperglycemia. In subsequent experiments, 1% CMC2.24 “normalized” wound-healing in the diabetics, whereas 1% curcumin was no more effective than 0.25% CMC2.24, and the latter remained 34% worse than normal. MMP-8 was increased 10-fold in the diabetic wounds and topically applied 1% (but not 0.25%) CMC2.24 significantly reduced this excessive collagenase-2; MMP-13/collagenase-3 did not show significant changes. Additional studies indicated efficacy of 1% CMC2.24 over more prolonged periods of time up to 30 days.

## 1. Introduction

Wound-healing in skin and other mucosal tissues involves a complex sequence of events including the clotting cascade, acute and chronic inflammation, reepithelialization, granulation tissue formation, wound contraction, and connective-tissue remodeling [1]. However, a number of genetic and acquired conditions, such as aging, malnutrition (e.g., vitamin C and protein deficiency), infection, hypoxia, and genetic diseases such as Ehlers-Danlos syndrome, can impair this reparative process [2]. Among these conditions, diabetes mellitus is a most common cause of impaired or nonhealing wounds. As an example, the clinical significance of long-term hyperglycemia is highlighted by alarming data showing that 85% of nonhealing diabetic foot ulcers ultimately require amputation [3, 4]. The “pathway to a chronic wound,” as outlined by authors of a recent study [5], focused on prolonged or chronic inflammation characterized by activation of macrophages (as well as accumulation of neutrophils) that resulted in elevated levels of proinflammatory cytokines, reactive oxygen species, matrix metalloproteinases (MMPs), and other neutral proteinases (e.g., elastase). This, coupled with a deficiency of endogenous proteinase inhibitors, all leads to “excessive matrix degradation, degradation of growth factors, and impaired epithelialization.”

In this regard, our discovery three decades ago that the tetracycline antibiotics (including doxycycline),* unexpectedly*, can inhibit host-derived MMPs such as the collagenases (MMP-1, MMP-8, and MMP-13) and gelatinases/type IV collagenases (MMP-2 and MMP-9) and by mechanisms* independent* of their antimicrobial activity [6, 7] resulted in a series of experiments on the effect of these MMP-inhibitors on wound-healing in several different animal models. These studies included the streptozotocin-induced type I diabetic rat and the ovariectomized rat model of postmenopausal osteoporosis. Both of these models produce aging-like changes in the collagen of the dermis, including skin atrophy associated with impaired collagen synthesis by fibroblasts, dramatic increases in collagenase and gelatinase activities, and excessive accumulation of older highly cross-linked collagen relative to newer more extractable collagen. All of these contribute, at least in part, to impaired wound-healing in skin [8, 9]. Using these and other models, we demonstrated that topical or systemic administration of tetracyclines, such as doxycycline, and the chemically modified nonantimicrobial tetracyclines or CMTs (aka COLs) can “normalize” wound-healing: (i) while having no effect on the severity of hyperglycemia in the diabetic rats [9]; (ii) in the presence of prolonged estrogen deficiency in ovariectomized aged rats [8]; and (iii) in sterile corneal ulcers (“corneal melts”) in rabbits and humans [10–12].

Based on our identification of the site on the tetracycline molecule responsible for its MMP-inhibitory properties, that is, the zinc-binding *β*-diketone moiety at carbons 11 and 12 [6, 7], we recently developed two new series of compounds that contain a similar zinc-binding site, but which are bicyclic rather than tetracyclic, namely, the chemically modified curcumins (CMCs) and the bis-aroylmethanes (BAMs). After testing the biologic activity of more than 30 of these novel compounds, using* in vitro*, cell, and tissue culture and* in vivo *rat and mouse models, we identified a “lead” compound, a phenylaminocarbonyl (triketonic rather than diketonic) curcumin, which showed the greatest efficacy in several models of diseases including diabetes, periodontitis, and osteoarthritis but demonstrated no evidence of toxicity when administered systemically* in vivo *either by oral intubation or by intraperitoneal injection or when tested in organ culture [13–16]. In the current study, we demonstrate that this novel compound, CMC2.24, administered either topically or systemically, can substantially improve wound-healing in skin of the severely hyperglycemic type I diabetic rat.

## 2. Materials and Methods

The details of the synthesis of CMC2.24 and its structure were described by us recently [14, 15]. The animal study was approved by the Institutional Animal Care and Use Committee (IACUC) of Stony Brook University.

In the first experiment, fifteen adult male Sprague-Dawley rats (body weight 300–325 g, Charles River Laboratories International, Inc., Wilmington, MA) were injected through the tail vein with either 10 mM citrated saline buffer pH 4.5 (nondiabetic controls, NDCs) or the same solution containing streptozotocin (STZ; ENZO Life Sciences, Inc., Plymouth Meeting, PA; 70 mg/kg body weight) to induce type I diabetes. The rats were then distributed into the five experimental groups described below (*n* = 3 rats/group). All rats were given unlimited access to food and water. Within 48 hours, the STZ-injected rats exhibited severely elevated glucose levels in their urine. Three weeks after inducing diabetes, the back skins of all the rats were shaved and a series of six standard wounds per rat, each 6 mm in diameter, were made using a surgical trephine. The following five experimental groups were established (in this initial experiment, treatment in all groups was for seven days; a longer-term study is described below in experiment 3): nondiabetic control (NDC) rats treated by daily topical application of white petrolatum jelly (“vehicle”); diabetic rats (D group) topically treated daily with vehicle alone; diabetic rats treated by daily topical application of either a 1% (D + 1% 2.24) or a 3% (D + 3% 2.24) suspension of CMC2.24 in the vehicle; and diabetics treated systemically by daily oral intubation of a 1 mL suspension of CMC2.24 in 2% carboxymethylcellulose at a dose of 30 mg/kg [16] over the 7-day treatment protocol (D + 30 mg 2.24). An “Elizabeth” collar (Lomir Biomedical Inc., Quebec, Canada) was placed around the neck of each rat during the initial seven days of healing to prevent rats from inflicting self-injury, for example, biting and scratching, and to prevent licking of the wounds. At the end of this time period, the six circular wounds per rat were clinically assessed by measuring with a caliper the diameter of the wounds in millimeters, blood samples were collected, the rats were sacrificed, and skin samples were dissected for histological/histochemical and biochemical assessment as described below. The techniques described above are essentially the same as those described by us previously using topically or systemically administered tetracyclines (doxycycline and the chemically modified nonantimicrobial tetracyclines or CMTs) in the diabetic male [9] and in the surgically induced menopausal (ovariectomized) female [8], both recognized rat models of impaired wound-healing in skin [8, 9].

On day seven after creating the standardized wounds, all of the animals were anesthetized, blood samples were collected for blood glucose (One Touch Ultra Glucometer; Johnson & Johnson, New Brunswick, NJ) and HbA1c (Bayer A1CNow Selfcheck, Sunnyvale, CA) measurements, and, after the procedures below were completed, the rats were sacrificed by CO2 inhalation.

Photographs were taken for clinical measurements to assess wound closure (18 wounds per experimental group). The percent reduction of the wound surface was calculated by measuring the diameter (in millimeters) of each wound before and after the treatment protocol.

Wound tissues on day 7 were excised from two sites per rat and pooled for biochemical analysis. Each pool of tissue was homogenized, extracted at 4°C with 5 M urea in 50 mM Tris-HCl buffer (pH 7.8) containing 0.2 M NaCl and 5 mM CaCl_2_ overnight, and then centrifuged for 1 hour at 11,000 ×g, as described by us previously [16]. The supernatants were dialyzed against the Tris-HCl, NaCl, and CaC1_2_ buffer, and the proteinases were partially purified by ammonium sulfate added to 60% saturation. The precipitated proteinases were analyzed by ELISA for collagenases MMP-8 (Sigma-Aldrich Life Sciences Inc., St. Louis, MO) and MMP-13 (TSZ Scientific LLC, Framingham, MA).

Biopsies of each of two wound sites, including surrounding nonwounded tissue, were taken and fixed in 10% neutral buffered formalin for 24 hours and then transferred to 50% ethanol prior to grossing, alcohol dehydration, xylene clearing, paraffin embedding, and sectioning. Five-micron sections were stained with H&E and Masson's Trichrome and the distance between wound margins was measured histomorphometrically using a calibrated ocular micrometer and confirmed image analysis [8, 9]. The last two wounds per rat were dissected, hydrolyzed, and analyzed for hydroxyproline as described below.

In the second experiment, using the same duration (7 days) of treatment as described in the first, eighteen male Sprague-Dawley rats (300–325 g body weight) were divided into six groups (*n* = 3 rats/group); some groups are different from those described in the first experiment—Group I: nondiabetic control rats treated topically with vehicle alone (NDC); Group II: diabetics treated with vehicle only (D); Groups III and IV: diabetics treated topically with either 0.25% or 1% CMC2.24 suspended in white petrolatum jelly, once daily for seven days; Groups V and VI: diabetics treated topically with either 0.25% or 1% curcumin (Sigma-Aldrich, St. Louis, MO), once daily for seven days. Photographs were taken for clinical observations every day. At sacrifice, wound tissues and blood were collected for biochemical and histological analysis as described above.

In the third experiment, a similar protocol was followed except that the twenty-four adult male rats were distributed into the following 6 experimental groups (*n* = 4 rats per group): Group I: NDC rats treated by daily application of white petrolatum jelly (“vehicle”) for 14 days; Group II: D rats treated with vehicle (14 days); and Group III: D rats treated with 1% CMC2.24 for 14 days.

The remaining twelve rats were distributed into the same three experimental groups; however the topical treatment of the NDC and D groups, with either vehicle alone or 1% CMC2.24, was carried out for 30 days after 3-week duration of STZ-induced diabetes.

At the end of the 14-day and 30-day treatment protocols, the physical measurements and histologic and histochemical assessments were the same as those described above for the 7-day experiment. In addition, for all three time periods, tissue samples from the 6 mm punch biopsies were hydrolyzed twice in 2 N NaOH at 120°C for 1 hour each time. 50 *μ*L aliquots of the skin tissue hydrolysates were then analyzed for hydroxyproline, an amino acid essentially found only in collagen [17, 18].

### 2.1. Statistical Analysis

Data were analyzed by Student's* t-*test. All values are expressed as the mean ± the standard error of the mean (SEM). *p* < 0.05 was considered statistically significant.

## 3. Results

**Figure 1:**
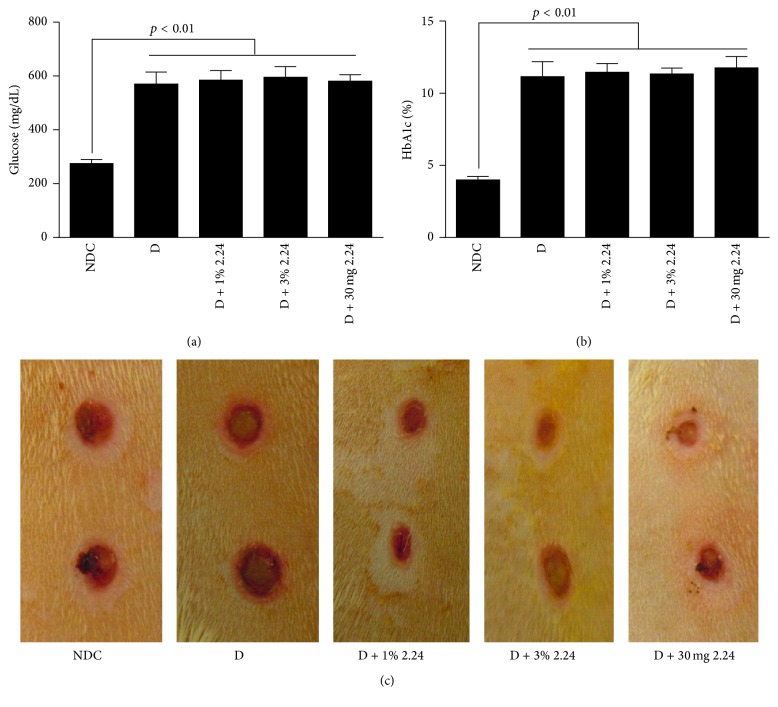
(a) Blood glucose and (b) HbA1c levels in the serum of normal (NDC) and diabetic (D) rats 4 weeks after STZ injection. The D rats were treated either by topical administration with vehicle (white petrolatum jelly) alone, or with a 1% or 3% suspension of CMC2.24 (D + 1% 2.24 or D + 3% 2.24, resp.), or by oral intubation with 30 mg/kg CMC2.24 suspended in 2% carboxymethylcellulose (D + 30 mg 2.24). The topical and systemic CMC2.24 treatments were administered daily during the final 7 days of the protocol, 3 weeks after inducing diabetes. Each value is the mean ± standard error of mean (SEM) of 3 rats per group. (c) The typical clinical appearance of the skin wounds from each of the five experimental groups 7 days after initiating the daily topical and systemic treatments. A *p* value ≤ 0.05 was considered statistically significant.

As shown in Figures 1(a) and 1(b), all four groups of D rats (including vehicle-only-treated D animals plus those D rats treated either topically or systemically with CMC2.24) exhibited significantly elevated blood glucose and hemoglobin A1c levels compared to the NDC rats, and none of the topical or systemic CMC2.24 treatments produced any detectable effect on the severity of hyperglycemia. However, despite this lack of benefit on blood glucose levels in the D rats, all of the CMC2.24 treatments (both topical and systemically administered) and, to a lesser extent, the curcumin treatments (Figures 3 and 4) enhanced wound-healing* in vivo*, as indicated in Figure 1(c) and as measured below.

### 3.1. Physical Measurements

**Figure 2:**
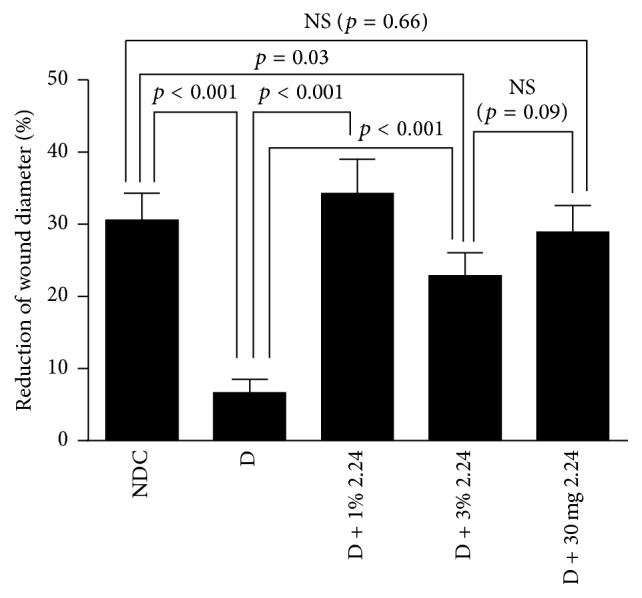
Healing, assessed clinically (% reduction in wound diameter), of skin wounds in normal (NDC) and diabetic (D) rats treated topically with vehicle alone or with 1% or 3% CMC2.24 or systemically with 30 mg/kg CMC2.24. Each value represents the mean ± SEM for 18 measurements (6 wounds per rat) per experimental group (*n* = 3 rats/group). NS: not statistically significant (*p* ≥ 0.05).

As expected, four weeks after inducing diabetes by STZ injection, the “clinical” appearance of the standardized skin wounds in these animals was consistent with impaired healing (Figure 1(c)). Note that, three weeks after inducing diabetes, the D rats were treated once each day, either topically or systemically, for an additional seven days with either CMC2.24 or vehicle alone (topical only); the NDC rats were only treated topically with vehicle alone. All of the D rats treated either topically with 1% or 3% CMC2.24, or systemically by oral gavage with 30 mg/kg of this compound, showed improvement and, in some groups, complete “normalization” of wound diameter was seen (Figures 1(c) and 2).

When the diameter of the wounds was measured using a caliper, the NDC rats, after 1 week of vehicle-alone treatment, showed a 30% reduction in wound size compared to the original 6 mm diameter lesion (Figure 2). However, experimental diabetes reduced wound closure by 77% (*p* < 0.001) compared to the NDC rats at this time period (Figure 2). Topical 1% CMC2.24 appeared to be the most effective treatment and significantly (*p* < 0.001) improved wound closure in the D rats and to a level which appeared to be about 10% better (*p* > 0.05; not significant) than that seen in the NDC group. Considering the other two treatments, orally administered CMC2.24 appeared to also “normalize” wound-healing in the D rats (D + 30 mg/kg versus NDC; *p* = 0.66) but was not significantly more effective than 3% CMC2.24 topically administered (D + 30 mg 2.24 versus D + 3% 2.24; *p* = 0.09). The latter treatment regimen, although seemingly the least effective of the three, appeared to improve wound-healing by 228% compared to the vehicle-treated D rats (D versus D + 3% 2.24; *p* < 0.001) but failed to equal the wound-healing characteristics of the NDCs (NDC versus D + 3% 2.24; *p* = 0.03).

### 3.2. A Comparison between Topically Applied CMC2.24 and Topical Curcumin

**Figure 3:**
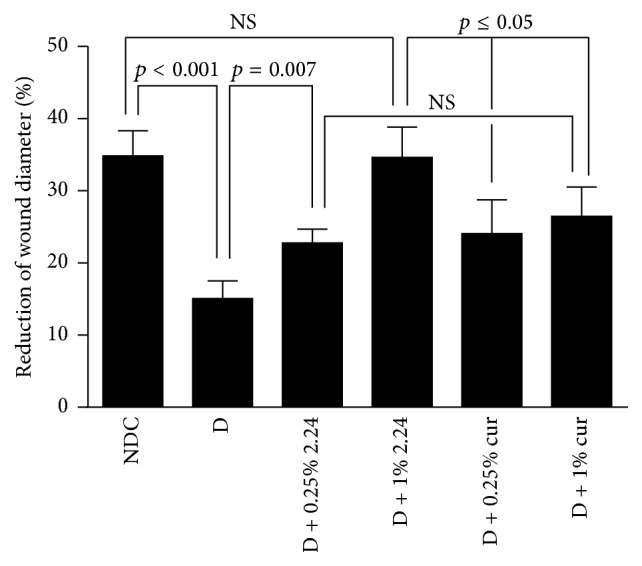
Histomorphometric assessment of healing (% reduction in wound diameter) of skin wounds in normal (NDC) and diabetic (D) rats treated with vehicle alone (D) and in D rats treated topically with either 0.25% CMC2.24 (D + 0.25% 2.24) or curcumin (D + 0.25% cur) or 1% CMC2.24 (D + 1% 2.24) or curcumin (D + 1% cur). Each value represents the mean ± SEM for 18 measurements per experimental group (3 rats/group).

In a second experiment similar to the one described above, the D rats, when assessed clinically, showed impaired wound-healing compared to the NDCs on day 7 after creating the circular 6 mm diameter wounds (Figure 3). In this experiment, the closure of the wounds was reduced by 57% (*p* < 0.001) in the D rats compared to the NDCs (Figure 3). CMC2.24 improved wound-healing in a dose-response fashion; 0.25% CMC2.24 improved wound-healing in the D rats by 53% (*p* = 0.007); however, closure was still 34% below normal. In contrast, 1% CMC2.24 appeared to completely “normalize” wound-healing clinically and was more effective than both 0.25% and 1% curcumin (*p* ≤ 0.05 for both curcumin concentrations versus 1% CMC2.24). The 1% concentration of curcumin was essentially as effective as 0.25% CMC2.24 in improving wound-healing in the D rats; both 0.25% CMC2.24 and 1% curcumin in the D rats exhibited about 30% impaired wound-healing compared to the NDCs (*p* > 0.05, 0.25% 2.24 versus 1% curcumin).

**Figure 4:**
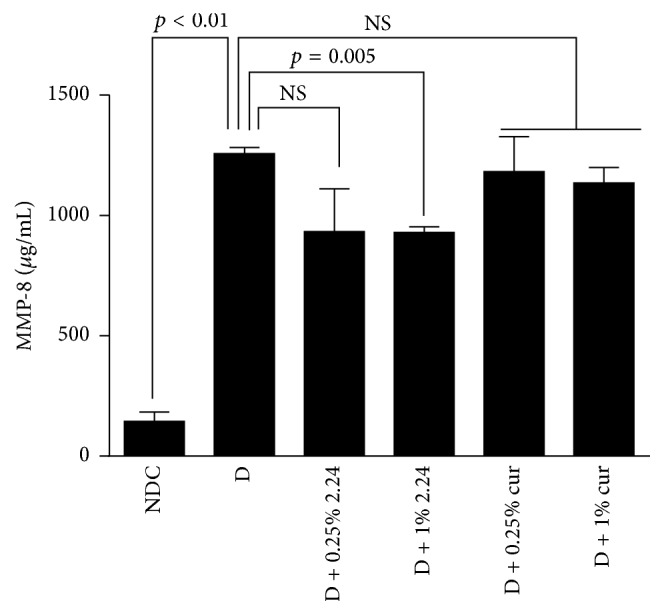
The concentration of MMP-8 (collagenase-2) in the wound extracts from the nondiabetic control (NDC) and diabetic (D) rats treated with vehicle alone or with 0.25% or 1% concentrations of CMC2.24 or curcumin. Each value represents the mean of 3 measurements per experimental group ± SEM.

Two different collagenases, MMP-8 (collagenase-2) and MMP-13 (collagenase-3), were assessed in the wound tissues on day 7 using ELISA techniques. In brief, MMP-13, which in rats is constitutive, analogous to MMP-1 in humans [19], did not show altered levels when comparing the NDCs to the various D groups (data not shown). In contrast, MMP-8 (also known as leukocyte-type collagenase) did show differences between the various groups of rats (Figure 4). A tenfold increase in MMP-8 (*p* < 0.01) was seen in the wound tissue from the D rats (vehicle-alone treatment) compared to the NDCs. Whereas neither the 0.25% nor 1% doses of curcumin, nor the 0.25% of CMC2.24, produced a statistically significant effect (*p* > 0.05) on MMP-8 levels in the diabetic wounds, the 25% reduction of MMP-8 by 1% CMC2.24 was statistically significant (*p* = 0.005) (Figure 4).

### 3.3. The Effect of CMC2.24 on Wound-Healing in Diabetic Rats: Histologic and Histochemical Assessment at Day 7

**Figure 5:**
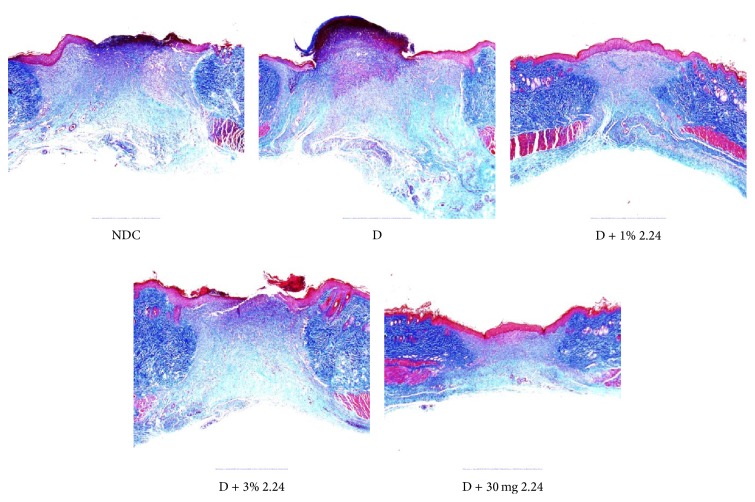
Masson's Trichrome staining of collagen (blue-green) in the healing dermis of the NDC and D groups indicates that (1) diabetes delays healing of the subepithelial connective tissue and (2) 1% CMC2.24 topically applied is more effective than the other treatments in helping “normalize” this aspect of wound-healing.

**Figure 6:**
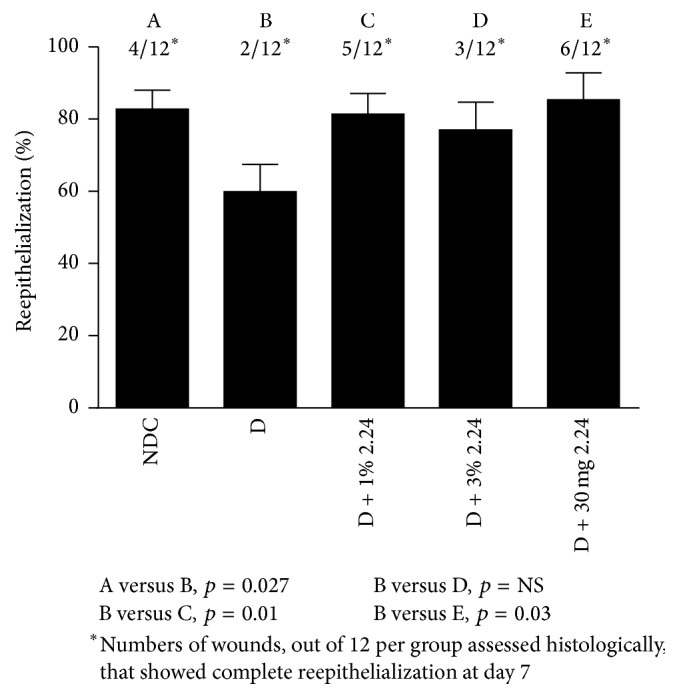
Histomorphometric analysis of reepithelialization of standardized skin wounds in nondiabetic control (NDC) and diabetic rats treated with vehicle (D) or with a 1% or 3% CMC2.24 topically applied (D + 1% 2.24 or D + 3% 2.24, resp.) or with 30 mg/kg CMC2.24 administered systemically by oral intubation (D + 30 mg 2.24). Each value (%) represents the mean closure of the epithelium, assessed histologically, for 12 histological slices per group ± SEM, and the letters, A–E, assign statistically significant differences between the experimental groups. The star (*∗*) symbol represents the numbers of wounds per group showing complete reepithelization on day 7.

The histologic assessment (Masson's Trichrome staining) of wound-healing in the different groups of rats (Figure 5) was generally consistent with the measurements described above. As shown in Figure 6, 4/12 of the NDC wound biopsies assessed histologically showed complete reepithelialization at day 7 after creating the lesions, whereas diabetes appeared to reduce the incidence of reepithelialization by 50%. Once again, two of the three CMC2.24 treatments, the 1% topically applied and the oral administration of 30 mg/kg body weight, essentially “normalized” this measure (i.e., reepithelialization) of wound-healing, whereas topical 3% 2.24 appeared slightly less effective. Consistent with this pattern, the histomorphometric assessment of wound reepithelialization demonstrated that the D group was 27% less than NDC (*p* = 0.027) and, compared to the D group, the efficacy of the three different treatments was as follows: 1% 2.24 (*p* = 0.01) > 30 mg/kg 2.24 (*p* = 0.03) > 3% 2.24 (*p* > 0.05); only the latter treatment was not statistically effective (*p* = NS; Figure 6) compared to the vehicle-treated D group. The histologic measurement of wound diameter (mm) on day 7 showed a similar pattern of change: the 38% larger diameter of the diabetic wounds, compared to the wounds in the NDC rats, was “normalized” by both the 1% topical and the systemic treatments with CMC2.24, whereas the 3% CMC2.24 did not significantly reduce the histologic wound diameter (data not shown).

**Figure 7:**
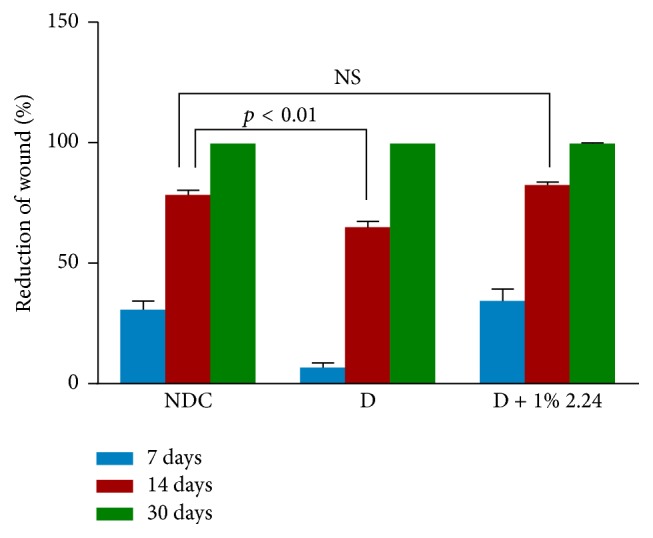
Healing (% reduction in diameter) of skin wounds in normal (NDC), diabetic vehicle-treated control (D), and diabetic topically treated with 1% CMC2.24 (D + 1% 2.24) rats, respectively, after 7 days, 14 days, and 30 days. Each value is the mean ± SEM for 18 measurements (3 rats/group) of 7 days and for 24 measurements (4 rats/group) of 14 days and 30 days.

**Figure 8:**
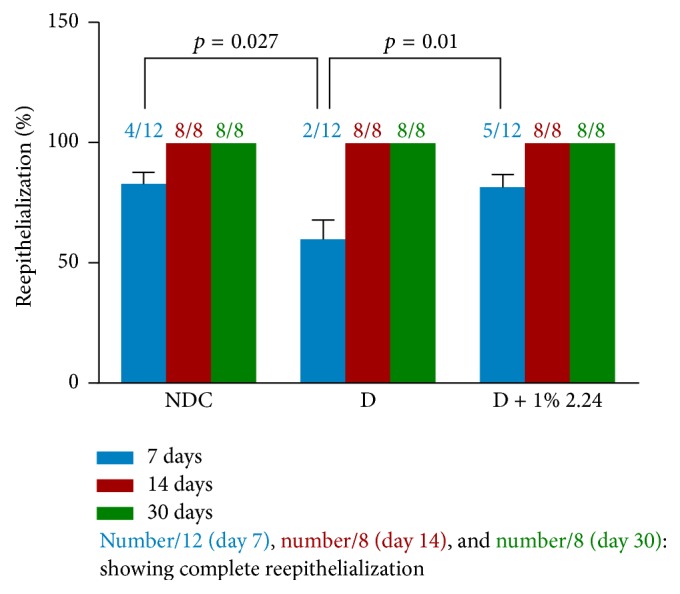
No difference in reepithelialization between normal and diabetic rats skin wound after day 14 administration of 1% topical CMC2.24 and vehicle alone. Day 7: each value represents the mean ± SEM of 12 histomorphometric analyses/group. Day 14: each value represents the mean ± SEM of 8 histomorphometric analyses/group. Day 30: each value represents the mean ± SEM of 8 histomorphometric analyses/group.

Based on the superior efficacy of the 1% CMC2.24 topical treatment, the longer-term effects of this regimen were also examined.  Figure 7 shows the progressive closure (% reduction of wound diameter) of the wounds in the different groups of rats at days 14 and 30, after creating the standardized lesions, compared to the day 7 data already described (see Figure 2). The pattern of closure for the different experimental groups at day 14 was similar to that for day 7; that is, for day 14 (Figure 7), the diabetic rats showed a 22% reduction in wound closure compared to the NDC rats (*p* < 0.01) which was completely returned to normal levels by topical CMC2.24 (NDC versus D + 1% 2.24; *p* > 0.05: NS). However, by day 30, all three experimental groups showed 100% reduction in the diameter of the standardized wounds (Figure 7). The pattern was different for reepithelialization of the wounds in the three experimental groups (Figure 8); that is, unlike the day 7 and day 14 data (see Figure 7) which demonstrated significant impaired wound closure in the vehicle-treated D rats compared to the NDCs, and normalization of closure at these two time periods in the D rats treated with CMC2.24, as shown in Figure 8, at days 14 and 30 all three groups (NDC, D, and D + 1% 2.24) showed complete (100%) reepithelialization of the standardized skin wounds assessed histologically. However the chronic status of the wounds in these various groups is not equivalent even after thirty days (when reepithelialization is complete) as is evident from the biochemical analysis of collagen content, now described.

### 3.4. Collagen Analysis

**Figure 9:**
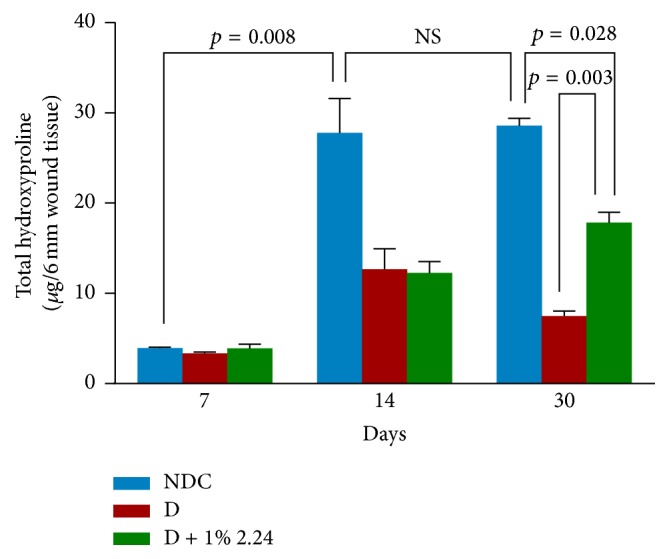
The effect of diabetes and CMC2.24 on hydroxyproline levels in skin wounds at 7, 14, and 30 days. Each value represents the mean ± SEM (3 rats/group) for 7 days and for 14 days and 30 days (4 rats/group).

The data in Figure 9 shows the collagen (assessed as hydroxyproline) levels in the wound tissues of the three experimental groups, NDC, D, and D + 1% 2.24 at days 7, 14, and 30 after creating the 6 mm diameter standardized lesions. In the nondiabetic control rats, the hydroxyproline levels in the healing wounds rapidly increased by over 500% (*p* = 0.008) by day 14 and remained at this level at day 30. The hydroxyproline levels in the vehicle-treated (D) and CMC-treated (D + 1% 2.24) diabetics increased much more slowly and did not reach the high levels in the NDC rats even at day 30. However, at this late time period, CMC2.24 treatment of the D rats wounds did increase the hydroxyproline levels by 140% (*p* = 0.003), compared to the vehicle-treated D rats, although the CMC2.24-treated diabetic wounds still showed hydroxyproline (collagen) levels 37% lower (*p* = 0.028) than those seen in the NDC rats on day 30.

## 4. Discussion

Matrix metalloproteinases (MMPs) are a multigene family of more than 25 zinc-dependent neutral proteinases involved in the degradation of extracellular matrix, nonmatrix constituents, and basement membranes. In addition, they process cytokines, chemokines, and growth factors, all of which participate in physiologic connective tissue turnover including bone remodeling and growth, embryonic morphogenesis, angiogenesis, and wound-healing [20, 21]. Dysregulation of MMPs is associated with a variety of inflammatory, apoptotic, and carcinogenic processes which mediate a substantial number of diseases such as (but not limited to) arthritis, atherosclerosis, periodontitis, and tumor invasion and metastasis [22–24].

MMPs play a role in all stages of wound-healing including inflammation, debridement of damaged connective tissue and reepithelialization in the early stages, and angiogenesis and connective tissue remodeling at later stages [25, 26]. Impaired wound-healing is a well-known complication of diabetes [1] which too often leads to chronic infection, amputation, and even death [3, 4], and virtually every aspect of wound-healing can be adversely affected by poorly controlled diabetes and hyperglycemia ranging from leukocyte dysfunctions and impaired reepithelialization to decreased fibroblast activity, suppressed collagen synthesis, and delayed regeneration. With regard to the MMPs, several studies have shown that the induction of experimental diabetes increases MMP expression and activity in tissues such as skin and gingiva [9, 26, 27] and that administration of nonantimicrobial tetracyclines (TCs) can reduce this excessive proteinase activity down to essentially constitutive/physiologic levels seen in nondiabetic/normal rats. Additional experiments demonstrated that both antimicrobial and nonantimicrobial TCs, applied either topically or systemically, “normalized” lesions in both diabetic and surgically induced menopausal rat models of impaired wound-healing [8, 9]. Recent studies have applied this approach in clinical trials involving nonhealing wounds in diabetic patients [5, 25], and topical 1% doxycycline ointment “healed the chronic diabetic ulcers better than the vehicle treatment” [25]. Mechanisms could include the dampening of the pathologically excessive levels of collagenases (MMP-1 and MMP-8) and gelatinases (MMP-2 and MMP-9) that have been observed in biopsies of diabetic foot ulcers, compared to the levels of these proteinases seen in normal posttraumatic wounds [28]. It should be recognized however, as previously discussed, that excessive inhibition of MMPs can be deleterious since these enzymes have physiologic functions [21, 22]. As one example, constitutive levels of MMP-8 have anti-inflammatory properties, which is consistent with the observation that MMP-8 knockout mice exhibit increased inflammation and delayed wound-healing [29].

In part, because of the limitations inherent in the use of TCs for wound-healing, including the potential for inducing the emergence of antibiotic-resistant bacteria and photosensitivity, we recently developed a new series of bicyclic rather than tetracyclic compounds with MMP-modulating activity associated, at least in part, with a similar *β*-diketone, zinc and calcium-binding site in the TC molecule. Initially, these bicyclic compounds included the use of the bis-aroylmethanes (BAMs) as well as a series of chemically modified curcumins (CMCs) [14, 15]. However, the initial “lead” compound, CMC2.5 (a triketonic 4-methoxycarbonyl curcumin), and, more recently, a newer and more potent compound, CMC2.24 (a phenylaminocarbonyl curcumin, also triketonic), showed superior efficacy in models of various collagenolytic diseases such as arthritis, periodontitis, and diabetes [13–16]. The current report is the first to describe the efficacy of CMC2.24 in an animal model of impaired wound-healing, namely, the severely hyperglycemic diabetic rat. In brief, assessment of the healing of standardized 6 mm diameter wounds in the dorsal skin of the diabetic rats, using clinical, histologic/histochemical, and biochemical measures, all indicates that either topical or systemic (by the oral route) administration of CMC2.24 significantly enhanced wound-healing and that this CMC is substantially more effective than the parent molecule, curcumin. It should be noted that several studies have described wound-healing benefits using natural curcumin [30, 31]. It should be also noted that CMC2.24 did not appear to significantly affect wound-healing in the nondiabetic control rats (data not shown).

Various aspects of the wound-healing process were significantly improved, even “normalized,” in this animal model of severe type I diabetes even though none of the treatments with this compound appeared to reduce the severity of hyperglycemia. In the early stages, delayed reepithelialization of the diabetic wounds was returned to normal by either topical or systemically administered CMC2.24. A later event, connective tissue regeneration in the dermis, also appeared to be significantly improved, if not completely “normalized,” in the diabetic rat wounds during CMC2.24 treatment; this beneficial effect was associated with increased collagen levels, assessed histochemically and by hydroxyproline analysis, and decreased pathologically excessive MMP activity. In this regard, MMP-8, which in the current study was generated in dramatically elevated levels during diabetes in the early inflammatory phase of wound-healing, was significantly reduced by CMC2.24 treatment; it is important to note that both MMP-8 (collagenase-2) and MMP-9 (gelatinase B) are released when polymorphonuclear leukocytes degranulate during the inflammatory response. In this regard, preliminary data in our lab indicates that this gelatinase/type IV collagenase, like MMP-8, was also increased by diabetes and that CMC2.24 treatment suppresses this excessive MMP during wound-healing—a similar pattern of change for this gelatinase was recently seen by us during experimental diabetes and its treatment with CMC2.24 in gingiva and skin [32]. In contrast, MMP-13 (collagenase-3), which in rats appears to be constitutive and analogous to MMP-1 (collagenase-1) in humans [19, 33], did not appear to be affected either by diabetes or by CMC2.24 during the 7-day treatment of the wounds (data not shown) indicating that this novel treatment selectively inhibits pathologically excessive MMPs but does not suppress constitutive MMPS needed for normal/physiologic connective tissue turnover. MMP-14 also appears to play a role in wound repair [34] and we have found that CMC2.24 is a potent inhibitor of this enzyme's activity [15]. However, whether CMC2.24 affects the expression of MMP-14 has not yet been determined.

This study also demonstrated both the short-term and long-term benefits of topical CMC2.24 treatment in this model of impaired wound-healing. In this regard, CMC2.24 accelerated the reepithelialization and closure of the wounds in the severely hyperglycemic rats in the short term (7 and 14 days after wounding). However, in the long term (day 30), this treatment also increased the collagen content of the dermis, which in the vehicle-treated diabetics remained at abnormally low levels. The excess MMP activity in the diabetic rats could also explain, at least in part, not only the reduced collagen levels in the wounds but also its reduced solubility (at 4°C) which is known to reflect excessive collagen cross-linking and aging [35, 36]. Of interest, CMC2.24 treatment was recently found to prevent this diabetes-induced defect [32]. Future studies are necessary to determine whether the improved level and quality (i.e., solubility) of the collagen in CMC2.24-treated diabetic rats reflects decreased MMP degradation of newly synthesized poorly cross-linked collagen (the preferred substrate for these MMPs), or an increase in synthesis of collagen (new collagen is characteristically more soluble and less cross-linked), or a combination of both.

Moreover, in a recent preliminary study, which has begun to assess the substantivity of this treatment, the improvements in wound-healing produced by the 2-week regimen of topical CMC2.24 were maintained for at least 2 more weeks after the treatment stopped, thus indicating that the severe diabetic state did not cause the deterioration of the 2.24-improved wounds after the topical treatment was terminated (data not shown).

Based on the current data, we propose the following working hypothesis: namely, this novel therapeutic compound “resolves” [37], rather than suppresses, the inflammatory phase of wound-healing, recognizing that inflammation is abnormally prolonged during the uncontrolled diabetic state in the absence of the therapeutic compound. In support of this hypothesis, recent preliminary studies in our lab on diabetic rats examined inflammatory cell functions in culture following* in vivo* treatment with CMC2.24 [38]. The results indicate that diabetes impairs the chemotactic activity of these cells, which leads to an impaired ability to combat infection and which contributes to prolonged inflammation [39]. However, administration of this novel compound “normalized” both the numbers of these leukocytes in the inflammatory exudate [38] and their chemotactic activity [39] preventing the prolongation of an incompetent inflammatory response and promoting more rapid wound-healing. These findings and conclusions are consistent with recent observations that proresolution lipid mediators improve delayed healing in a mouse model of type 2 diabetes [40].

## 5. Conclusion

Impaired wound-healing in skin and other tissues is a major, sometimes fatal, complication of diabetes mellitus. The current study demonstrated that a 1% suspension (better than 0.25% or 3%) of a novel chemically modified curcumin, a phenylaminocarbonyl triketonic curcumin (CMC2.24), was more effective, topically applied to standardized wounds in the skin of STZ-diabetic rats, than diketonic natural curcumin in accelerating reepithelialization and collagen replacement in the presence of unaffected severe hyperglycemia. Systemically administered CMC2.24 (oral gavage, 30 mg/kg qd) showed similar efficacy as the topical formulation, based on clinical, histologic, and biochemical (hydroxyproline/collagen; matrix metalloproteinases/MMPs) evidence, assessed over time periods from 7 to 30 days. Future clinical studies are needed to assess the potential of this novel compound in the management of impaired wound-healing in diabetic patients.
